# Protein profile in *Aspergillus nidulans* recombinant strains overproducing heterologous enzymes

**DOI:** 10.1111/1751-7915.13027

**Published:** 2018-01-08

**Authors:** Mariane Paludetti Zubieta, Fabiano Jares Contesini, Marcelo Ventura Rubio, Any Elisa de Souza Schmidt Gonçalves, Jaqueline Aline Gerhardt, Rolf Alexander Prade, André Ricardo de Lima Damasio

**Affiliations:** ^1^ Department of Biochemistry and Tissue Biology Institute of Biology University of Campinas (UNICAMP) Campinas SP Brazil; ^2^ Department of Internal Medicine Faculty of Medical Sciences University of Campinas (UNICAMP) Campinas SP Brazil; ^3^ Department of Microbiology and Molecular Genetics Oklahoma State University Stillwater OK USA

## Abstract

Filamentous fungi are robust cell factories and have been used for the production of large quantities of industrially relevant enzymes. However, the production levels of heterologous proteins still need to be improved. Therefore, this article aimed to investigate the global proteome profiling of *Aspergillus nidulans* recombinant strains in order to understand the bottlenecks of heterologous enzymes production. About 250, 441 and 424 intracellular proteins were identified in the control strain Anid_pEXPYR and in the recombinant strains Anid_AbfA and Anid_Cbhl respectively. In this context, the most enriched processes in recombinant strains were energy pathway, amino acid metabolism, ribosome biogenesis, translation, endoplasmic reticulum and oxidative stress, and repression under secretion stress (RESS). The global protein profile of the recombinant strains Anid_AbfA and Anid_Cbhl was similar, although the latter strain secreted more recombinant enzyme than the former. These findings provide insights into the bottlenecks involved in the secretion of recombinant proteins in *A. nidulans*, as well as in regard to the rational manipulation of target genes for engineering fungal strains as microbial cell factories.

## Introduction

Numerous efforts have been made to develop strategies that can supply the enzyme market, as well as those aimed at reducing its costs. Some of these include selecting an appropriate enzyme source and optimizing enzyme properties and secretion. Carbohydrate‐active enzymes (CAZymes) are industrially relevant biocatalysts capable of degrading plant cell wall biomass. The most important secreted enzymes related to plant cell wall decomposition are cellulases, hemicellulases and auxiliary enzymes (Levasseur *et al*., 2013; Lombard *et al*., 2014). These enzymes have been applied in plant biomass hydrolysis to produce second‐generation ethanol and several other high‐added value products (Segato *et al*., 2014; Goldbeck *et al*., 2016). Some of these enzymes include α‐L‐arabinofuranosidases (EC 3.2.1.55) that belong to glycoside hydrolase families 51, 54 and 62 (GH51, 54 and 62). These accessory enzymes hydrolyse α‐L‐arabinofuranosidic linkages and have different applications (Rémond *et al*., 2004). Another important group of enzymes corresponds to the cellobiohydrolases (EC 3.2.1.176), which hydrolyse cellulose chains by removing cellobiose and play a key role in cellulose degradation when combined with endoglucanases and lytic polysaccharide monooxygenases (Segato *et al*., 2012a; Vermaas *et al*., 2015).

*Aspergillus* spp. is an ascomycete that secretes remarkable quantities of proteins. *Aspergillus nidulans* is a genetic model that has been studied for the heterologous production of different CAZymes with promising results (Segato *et al*., 2012b). In addition, *Aspergillus* spp. is a suitable cell factory to produce heterologous enzymes from eukaryotic organisms recognizing and correctly processing introns (Jeenes *et al*., 1991) and has a tight regulation system for the N‐glycosylation of proteins (Larkin and Imperiali, 2011), including CAZymes (Rubio *et al*., 2016).

In spite of the fact that filamentous fungi present several advantages compared to other microorganisms due to the high level of protein production/secretion, heterologous protein production is far from optimal levels and there is still a need for improvements (Nevalainen and Peterson, 2014). Currently, heterologous production of certain proteins is considerably lower than the levels obtained for homologous proteins (Gouka *et al*., 1997). In this context, how fungal cells adapt to protein overexpression has not yet been significantly established, because protein secretion pathway involves more than 300 genes (Liu *et al*., 2014).

Many strategies have been studied to improve heterologous protein production by filamentous fungi, which include deleting genes that encode for proteases (Zhang *et al*., 2014; Landowski *et al*., 2015), deleting lectin‐like ER‐Golgi cargo receptors (Hoang *et al*., 2015) and co‐expressing chaperones with the heterologous protein of interest (Conesa *et al*., 2002). Therefore, more profound knowledge regarding the intracellular protein profile could shed light on the drawbacks and bottlenecks of heterologous protein production. For this purpose, an intracellular proteomic approach could be efficient to perform an overall analysis of the proteins involved in the complex regulatory circuits, which frequently result in different intracellular stress conditions.

Investigating individual genes and changes in the genome is not the best option to unveil the main bottlenecks in heterologous protein secretion (Nevalainen and Peterson, 2014). However, understanding the complex interactions of important proteins and genes, as well as how they are regulated, is a more promising option. For the purposes of our research, we applied mass spectrometry‐based proteomic approaches to understand how *A. nidulans* adapts to the high expression and production of heterologous proteins by analysing intracellular proteomes. We compared three *A. nidulans* strains, along with an empty plasmid‐transformed strain and two heterologous strains producing GH51 arabinofuranosidase (*abfA*) and GH7 cellobiohydrolase (*cbhI*) – both genes were isolated from *Aspergillus fumigatus*. Although there have already been several studies conducted on the proteomic analysis of different *Aspergillus* species, to the best of our knowledge there are no studies that have investigated the intracellular protein profile of *A. nidulans* strains overexpressing heterologous proteins.

## Results and discussion

### The *abfA* and *cbhl* genes were highly expressed in recombinant strains

The aim of this research was to analyse the intra‐ and extracellular proteome of two recombinant strains Anid_AbfA and Anid_Cbhl, which produce heterologous arabinofuranosidase (GH51‐AbfA) and cellobiohydrolase (GH7‐Cbhl) respectively.

**Figure 1:**
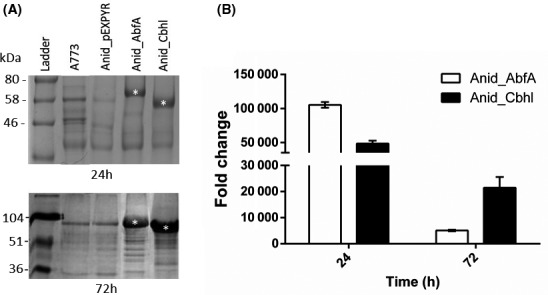
Secretion of total proteins by *Aspergillus nidulans* strains. The *A. nidulans* strains A773, Anid_pEXPYR (carrying an empty pEXPYR vector), Anid_AbfA and Anid_Cbhl expressing α‐L‐arabinofuranosidase and cellobiohydrolase, respectively, were grown on minimum media containing 2% maltose for 24 h and 72 h at 37 °C. (A) Ten micrograms of secreted proteins was resolved by Coomassie blue‐staining SDS‐PAGE gel. The strains A773 and Anid_pEXPYR were used as a control in this experiment. Asterisks (*) indicated the recombinant proteins. (B) qPCR of the recombinant genes was calculated by the relative standard curve method. The expression of genes *abfA* and *cbhl* was normalized using the gene *tubC* (tubulin) as reference. MM: molecular marker.

Initially, we evaluated the profile of secreted proteins following 72 h of maltose induction. The strains Anid_AbfA and Anid_Cbhl secreted large quantities of proteins, although Anid_Cbhl accumulated a higher amount of recombinant protein than Anid_AbfA (Fig. 1A). To evaluate these strains at the transcriptional level, the heterologous gene expression was quantified by qPCR. The *abfA* and *cbhl* genes were highly expressed in recombinant strains, Anid_AbfA and Anid_Cbhl respectively (Fig. 1B). This result indicates that the heterologous genes *abfA* and *cbhl* were efficiently transcribed, translated and secreted by *A. nidulans*.

**Figure 2:**
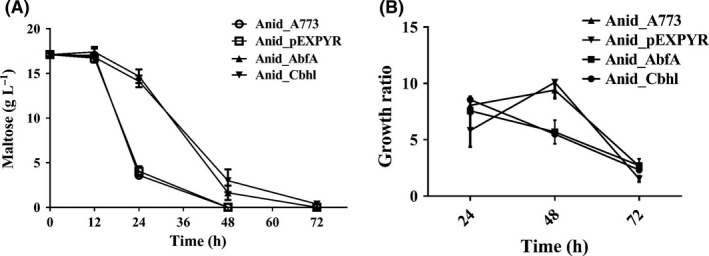
Analysis of *Aspergillus nidulans* growth. Spores solution was inoculated in 30 ml of minimum medium (MM) supplemented with 2% (m/v) maltose. (A) After various time points at 37 °C, the supernatant was separated from the culture medium by gauze filtration and maltose content was measured by HPLC. (B) The mycelia of *A. nidulans* strains were dried overnight at 105 °C for measure of the dry weight. Each bar represents the mean and the standard deviation of values from three independent experiments.

To determine the point of time for intracellular proteomic profiling, the growth of all strains on 2% maltose was evaluated. A faster uptake of maltose was observed for the control strains, A773 and Anid_pEXPYR, compared with the recombinant strains. After 24 h, the control strains consumed over 80% of the maltose, while only a small percentage of maltose was consumed by strains overexpressing heterologous genes (~23%). At 48 h, no maltose was present in the medium of either control strains (Fig. 2A). The slower consumption of maltose in the recombinant strains reflects slower growth ratio (Fig. 2B). In several protein expression systems that use fungi as cell factories, slow growth conditions may ensure that cells allocate sufficient resources to recombinant protein production (Liu *et al*., 2014). Considering this, we established 24 h as the point of time for our proteomic analysis, which is when there is some maltose remaining to support the growth of all strains.

### The intracellular proteome of recombinant strains is closely related

**Figure 3:**
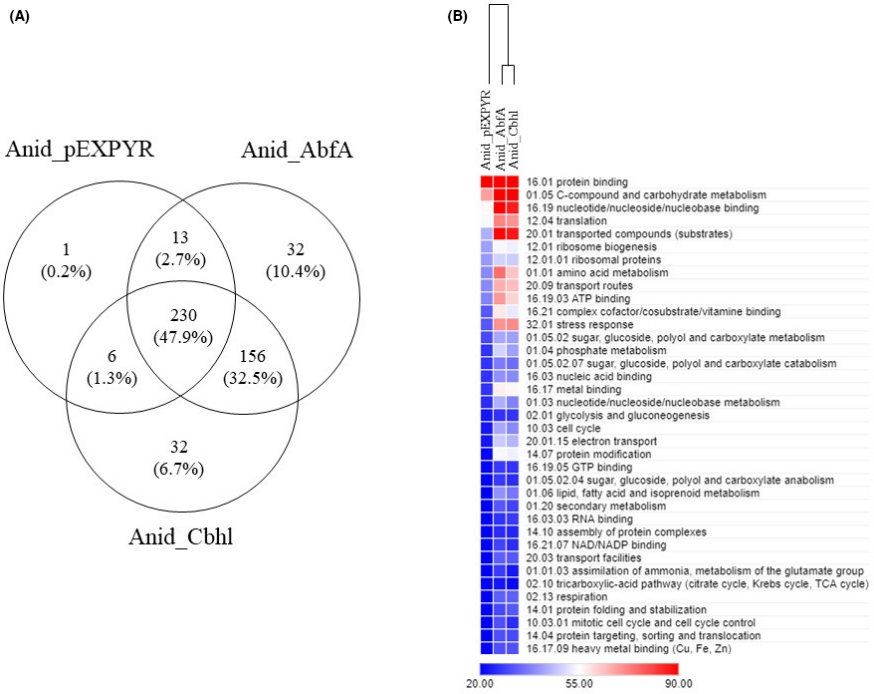
Abundance and functional analysis of intracellular proteins. The strains were grown on 2% maltose minimum medium for 24 h. (A) Venn diagrams represent the number of total proteins found in the intracellular proteome of each strain as well as the overlaps among groups. (B) A heat map of the 480 proteins categorized by MIPS FunCat (see Table S2) and the scale indicates the number of proteins found in each category. The intracellular proteomes were clustered based on their total spectra profiles.

Intracellular proteins were assessed by LC‐MS/MS. The total number of proteins identified in the strains Anid_pEXPYR, Anid_AbfA and Anid_Cbhl were 250, 441 and 424 respectively (Table S1). Around 47.9% of the 480 proteins identified were common to all strains, 32.5% were exclusively found in the recombinant strains Anid_AbfA and Anid_Cbhl, and 0.2% was exclusively found in the control strain (Anid_pEXPYR) (Fig. 3A). The results show that the protein profile of recombinant strains is especially closely related, and it is likely that this profile represents a pattern of cell response to heterologous proteins production in *A. nidulans*.

Additional analysis was performed using FunCat (The Functional Catalogue) annotations. Most of the proteins were annotated into the functional category of protein binding and C‐compound and carbohydrate metabolism (Fig. 3B). Within the protein binding category, the elongation factor 1‐α (AN4218) was present in higher quantities. This elongation factor, homologous to the *Saccharomyces cerevisiae* TEF‐1 and TEF‐2, delivers aminoacylated tRNA to the A‐site of ribosomes to elongate nascent polypeptides during protein translation. Due to its role in the cell, TEF‐1 is usually found at high levels in *S. cerevisiae*, which represents 5% of the total soluble protein pool (Thiele *et al*., 1985). Due to the strength of the promoter activity, several studies have reported the use of the *tef* promoter in protein production systems (Kitamoto *et al*., 1998; Magalhães *et al*., 2013).

The functional categories nucleotide/nucleoside/nucleobase binding, transported compounds, ATP binding, amino acid metabolism, translation, transport routes and stress response were further overrepresented, mainly in the recombinant strains. In Anid_AbfA and Anid_Cbhl, oxidoreductases were found more abundantly, namely 6‐phosphogluconate dehydrogenase (AN3954), aldehyde dehydrogenase (AN0554), mitochondrial malate dehydrogenase (AN6717) and NADP‐specific glutamate dehydrogenase (AN4376). Cultivation on maltose was previously associated with the presence of large amounts of oxidoreductases synthesized by fungi. The secretome analysis of *Aspergillus niger* grown on maltose, xylose and sorbitol, showed larger quantities of oxidoreductases on the maltose, such as superoxide dismutase and peroxiredoxin (Lu *et al*., 2010; Oliveira *et al*., 2011).

### Biological processes altered in *A. nidulans* recombinant strains

In order to determine a biological response profile in regard to heterologous protein production, we performed a comprehensive analysis of the intracellular proteins by total spectra. The proteins were classified as more or less abundant according to the number of total spectra relative to the control strain.

**Figure 4:**
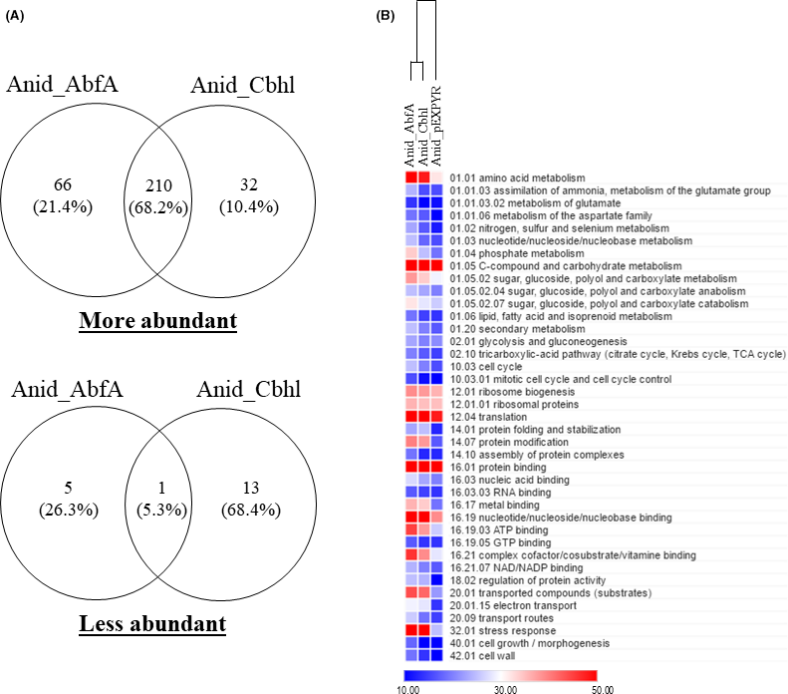
Abundance and functional analysis of intracellular proteins. The strains were grown on minimum medium and 2% maltose for 24 h. (A) Venn diagrams represent the number of more and less abundant proteins relative to *A. nidulans* Anid_pEXPYR strain. (B) A heat map of all proteins (see Table S3). MIPS FunCat categorization of the 308 more abundant proteins and 19 less abundant proteins common to Anid_AbfA and Anid_Cbhl strains. The scale indicates the number of proteins found in each category. The intracellular proteomes were clustered based on their total spectra profiles.

Overall, 276 (84%) and 242 (74%) proteins were more abundant in Anid_AbfA and Anid_Cbhl respectively. Almost all the proteins found in recombinant strains were classified as more abundant, while 6 (2%) and 14 (4%) proteins were classified as less abundant in Anid_AbfA and Anid_Cbhl respectively. The protein profiles were similar between recombinant strains, especially within the more abundant proteins group (Fig. 4A and Table S2). According to FunCat annotations, most of the more abundant proteins were related to protein binding (105 proteins in Anid_AbfA and 94 proteins in Anid_Cbhl). The second enriched functional category was C‐compound and carbohydrate metabolism with 75 proteins in Anid_AbfA and 65 proteins in Anid_Cbhl. Other categories were enriched such as translation, nucleotide/nucleoside/nucleobase binding, amino acid metabolism and stress response. In the group of less abundant proteins, 19 proteins were annotated (Fig. 4B). Hereafter, we described the main functional processes altered in the recombinant strains Anid_AbfA and Anid_Cbhl.

#### Energy pathway

About 190 proteins were annotated into the functional energy pathway category, comprising C‐compound and carbohydrate metabolism and amino acid metabolism. At least seven proteins that are directly involved in glycolysis or tricarboxylic acid (TCA) cycle (AN2436, AN5525, AN5746, AN2875, AN8041, AN6717, AN6499 and AN1246) were more abundant in the recombinant strains. In agreement with our results, there was a reported increase in the TCA cycle flux during Fab‐fragment antibody 3H6^12^ production in *Pichia pastoris*, indicating an increased energy demand. In yeasts, this increased energetic cost in recombinant protein production can be related to protein refolding and secretion (Dragosits *et al*., 2009; Tyo *et al*., 2012).

Enzymes of the pentose phosphate pathway (PPP) were also more abundant such as transketolase (AN0688) and transaldolase (AN0240). PPP is responsible for generating NADPH and pentoses as well as ribose 5‐phosphate, a precursor for nucleotides synthesis. NADPH is particularly necessary for biosynthesizing amino acids for use as building blocks for proteins. The demand for recombinant protein biosynthesis requires larger quantities of NADPH that become insufficient to support the normal *A. nidulans* growth, which may explain why the recombinant strains showed a lower growth rate than the control strains. An increased PPP flux was also observed for *A. niger* and *Aspergillus oryzae* producing fructofuranosidase and amylase respectively (Pedersen *et al*., 1999; Driouch *et al*., 2012). Furthermore, the activity of PPP was highest in *S*. *cerevisiae* during the expression phase of heterologous protein, β‐galactosidase, thereby resulting in an improvement of the foreign protein expression and cellular ATP yield (Jin *et al*., 1997).

The enrichment of TCA and PPP pathways confirms the higher energy requirement during heterologous protein production in filamentous fungi. This result shows that, among the various reactions of the central metabolism, these two pathways play a central role for recombinant protein production in fungal cells.

#### Amino acid metabolism, ribosome biogenesis and translation

Proteins related to amino acid metabolism were more abundant in Anid_AbfA and Anid_Cbhl, primarily enzymes with predicted role in methionine, alanine, aspartate, glutamine and glutamate metabolism such as methionine synthase (AN4443), NADP‐linked glutamate dehydrogenase (AN4376), alanine transaminase (AN1923), adenosylhomocysteinase (AN1263) and S‐adenosylmethionine synthetase (AN1222). Amino acid supplementation of the growth medium was shown to partially unburden cellular metabolism during recombinant protein production in yeast (Görgens *et al*., 2005; Heyland *et al*., 2011). In *S. cerevisiae*, adding a balanced mixture of the preferred amino acids, Ala, Arg, Asn, Gln and Gly, improved recombinant xylanase production (Görgens *et al*., 2005).

Translation efficiency is usually the first concern when designing expression systems for heterologous protein production. We found that the synthesis of proteins involved in translation was significantly more abundant during recombinant protein production. Translation elongation factors AN1162, AN4218, AN6330 and AN6563 were found more abundantly as well as ribosome structural proteins such as AN8176 and AN3413. During previous transcriptome studies, amino acid metabolism‐related genes were downregulated in recombinant strains, which might be due to the slower growth during the sampling period or the feedback inhibition of amino acid biosynthesis from ER stress overloaded (Liu *et al*., 2014). Transcription of genes encoding for translational and ribosomal proteins is also coordinated with and is essential for cell growth, proliferation and differentiation. ER stress induction with DTT (dithiothreitol) reduced the growth rate of *A. nidulans* chemostat culture concomitantly with the downregulation of 34 (81%) ribosomal genes (Sims *et al*., 2005).

Maltose consumption was lower for recombinant strains (Fig. 2A). Overall, this shows that Anid_AbfA and Anid_Cbhl are slow‐growing strains due to ER stress as well as other *Aspergillus* recombinant strains (Liu *et al*., 2014). Moreover, the lower growth ratio along with upregulation of amino acid metabolism, ribosome biogenesis and translation process in Anid_AbfA and Anid_Cbhl may reflect the increased energy demand required for heterologous protein production.

#### Endoplasmic reticulum stress

The unusually high and non‐physiological rates of recombinant protein production in filamentous fungi drive cells to an ER stress condition (Saloheimo *et al*., 1999). We detected proteins related to ER stress including chaperones (BipA, SgdE, Hsp104, Hsp70, Hsp90), foldases (Pdi, TigA, AN8605 and AN3814) and calnexin (AN3592) in both recombinant strains, showing that ER stress was actively turned on. These proteins were classified into response to stress, response to chemical and protein‐folding categories. The overproduction of several homologous and heterologous proteins in *Aspergillus* strains results in a condition called *unfolded protein response* (UPR), which is characterized by the accumulation of incorrectly folded proteins or delayed folding during ER (Heimel, 2015). The basic leucine zipper (bZIP) transcription factor HacA is responsible for the transcriptional induction of UPR‐target genes, which include ER‐localized molecular chaperones and folding, components of the ER‐associated degradation system, and other proteins acting at various stages of secretion, the purpose of which being disperse unfolded proteins accumulated during the ER (Pakula *et al*., 2003; Sims *et al*., 2005). The chaperones Hsp70, BipA and SgdE are useful to achieve an initial folding of nascent polypeptide in the ER (Mayer and Bukau, 2005). Hsp90 is responsible for folding and maintaining client proteins, transcriptional and post‐transcriptional processes and activation of signal transducers (Zuehlke and Johnson, 2010), while Hsp104 is responsible for reactivating denatured and aggregated proteins (Grimminger‐Marquardt and Lashuel, 2009). Calnexins are lectin chaperones that undergo releasing and re‐binding cycles until the glycoprotein achieves its native conformation, after which the protein is released for secretion into the distal secretory pathway (Molinari *et al*., 2003).

Foldases, peptidyl‐prolyl cis or trans isomerase (PPI) (cyclophilins), and protein disulfide isomerase (PDI) facilitate the folding of several proteins by catalysing the isomerization of prolyl peptide bonds between its cis and trans forms and formation and isomerization of disulfide bonds for proper folding respectively (Schönbrunner and Schmid, 1992). Induction of several UPR genes was detected in *A. niger* producing recombinant tissue plasminogen activator (t‐PA), such as *bipA*,* pdiA* and *pdiB* that are HSP70‐family chaperones (Guillemette *et al*., 2007). During the transcriptome analysis of recombinant bovine chymosin and α‐amylase production by *Aspergillus* strains, chaperones and foldases genes were also upregulated (Sims *et al*., 2005; Liu *et al*., 2014).

Several studies have reported that inducing the UPR genes in recombinant strains alleviates ER stress and may result in an improved secretion of a target protein. Thus, several strategies have been followed, such as the overexpression of protein disulfide isomerases or heat‐shock proteins acting as chaperones, but the results have been highly variable. The overexpression of chaperone BIPA and protein disulfide isomerase (PDIA) increased the secretion of a single‐chain antibody fragment in *S. cerevisiae* by twofold and eightfold respectively (Shusta *et al*., 1998). In *S. cerevisiae*, the overexpression of *bipA* increased the amount of extracellular prochymosin more than 20‐fold, but the secretion of thaumatin was not significantly stimulated (Harmsen *et al*., 1996). In *A. niger*, the expression of the activated form of the transcription factor *hacA* enhanced the production of *Trametes versicolor* laccase by sevenfold and bovine preprochymosin by 2.8‐fold (Valkonen *et al*., 2003).

BIPA (AN2069) and PDIA (AN7436) were twofold and threefold more abundant in Anid_Cbhl than Anid_AbfA. These data can be related to different intensity levels of UPR in the recombinant strains, which has already been demonstrated for DTT‐treated yeasts (Pincus *et al*., 2010). We suggest that UPR is more intense in Anid_Cbhl, resulting in the improvement of the cell protein‐folding capacity due to a highest induction of chaperones and foldases.

Chaperonins (AN2918 and AN5713) were additionally more abundant at the 24h point of culture in both recombinant strains. Chaperonins are essential to mediate the ATP‐dependent cellular protein folding in eukaryotes. Its interactome plays an important role in the folding or assembly of a range of proteins linked to the central and essential cellular processes, such as cytoskeleton assembly, cell‐cycle regulation and chromatin remodelling (Yam *et al*., 2008). In humans, the chaperonin TRiC/CCT regulates HSF1, an evolutionarily conserved transcription factor that protects cells from protein‐misfolding‐induced stress and apoptosis (He *et al*., 2015). However, we found no reports in the literature linking UPR and chaperonin‐dependent transcription. In general, the presence of chaperonins in recombinant cultures may be involved in helping cells to restore protein‐folding homeostasis.

In this study, our results suggest that producing heterologous proteins induced UPR in the recombinant strains due to an overload in the secretory pathway. This signalling network alleviates ER stress, promotes cell survival and adaptation and restores cellular folding homeostasis (Kozutsumi *et al*., 1988; Hollien, 2013).

#### Oxidative stress

The high secretion of recombinant proteins coordinately induced the production of proteins involved in oxidative stress in Anid_AbfA and Anid_Cbhl. We identified catalase (*catB*), thioredoxin (*trxA*), protein disulfide oxidoreductase activity (*ero1*) and aldehyde dehydrogenase (*aldA*) belonging to this group (Table S2). However, Anid_Cbhl showed a higher number of total spectra for *trxA* and *aldH* than Anid_AbfA. In the fungal platforms for protein production, protein folding is a crucial step of the secretory pathway, as a correct folding determines whether the newly synthesized protein will be targeted for secretion; otherwise, it will be assigned for ER‐associated degradation (ERAD). In many cases, protein folding includes disulfide bond formation, which in eukaryotes is managed by the coordinated action of PDIs and Ero1, using molecular oxygen as the terminal electron acceptor, and generating reactive oxygen species (ROS) (Tu *et al*., 2000). Heterologous proteins require an overall ER folding capacity, resulting in misfolded endogenous proteins that can limit the efficiency of protein synthesis. Furthermore, non‐native disulfide bonds are frequently formed during this process, which must then be broken down and subsequently rearranged to form the correct ones, thereby resulting in ROS accumulation and damage to biological macromolecules, such as DNA, lipids and proteins (Tyo *et al*., 2012). Thus, an increased demand for protein folding and disulfide bonds activates oxidative stress defence, which includes the upregulation of catalases and thioredoxins. Oxidative stress was previously described in yeast, which supports an increase in the protein production capacity during batch fermentations (Tyo *et al*., 2012; Martínez *et al*., 2016). Here, the higher total spectra of oxidoreductases in the recombinant strains could alleviate the oxidative stress and improve protein production.

#### Repression under secretion stress (RESS) – the secretion of carbohydrate‐active enzymes is reduced in recombinant strains

RESS is a transcriptional feedback mechanism that has been shown in filamentous fungi (Pakula *et al*., 2003; Al‐Sheikh *et al*., 2004). The expression of gene encoding to endogenous secreted proteins is downregulated by the UPR. Subsequently, the cargo load in ER decreases and accelerates the recovery of cell homeostasis (Pakula *et al*., 2003). This mechanism could occur in wild‐type and heterologous expression systems that have a high target protein flux through ER (Guillemette *et al*., 2007), such as Anid_AbfA and Anid_Cbhl.

To investigate RESS mechanism in the recombinant strains, we performed a secretome analysis to verify whether this mechanism occurs in *A. nidulans* recombinant strains. The secretion of some CAZymes was reduced in the recombinant strains. Around 86% of the secreted proteins significantly less abundant in the recombinant strains were CAZymes, such as endo‐arabinanase (AN8007), feruloyl esterase (AN5267), pectate lyase (AN7646 and AN8453), pectin‐methyl esterase (AN3390), β‐1,4‐endoxylanase and β‐glucosidase (AN2828) (Table S3). Previous transcriptome profiling of *N. crassa* cultures on cellulose showed that the lignocellulase genes were downregulated, suggesting the presence of RESS that may be limiting lignocellulase synthesis (Fan *et al*., 2015).

**Figure 5:**
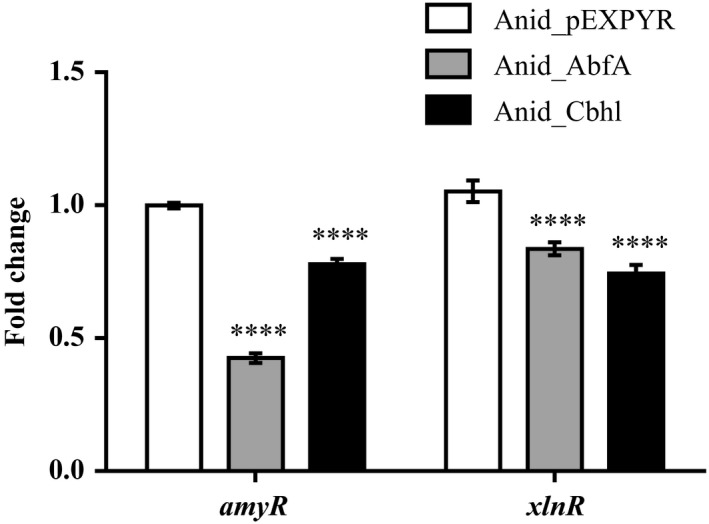
Evidence of *repression under secretion stress* (RESS) in *Aspergillus nidulans* recombinant strains. Transcriptional level of *amyR* and *xlnR* genes in Anid_AbfA and Anid_Cbhl was compared to the expression level in control strain (Anid_pEXPYR). Gene expression levels were normalized (ΔΔCT analysis) to the endogenous gene *tubC* (tubulin). Data were analysed using one‐way‐ANOVA with Bonferroni's post hoc test (****P < 0.0001).

We checked the level of transcripts of *amyR* and *xlnR*, two transcriptional regulators involved in the control of amylases and CAZymes‐encoding genes, respectively, because these regulators are downregulated under RESS (Carvalho *et al*., 2012; Zhou *et al*., 2014). *amyR* and *xlnR* transcripts were detected at lower levels in the recombinant strains when compared to the control strain. It is likely to relate the downregulation of these transcription factors to the reduction in CAZymes and amylases secretion (Fig. 5). In addition, downregulation of *amyR* may be related to lower growth of recombinant strains due to participation in maltose transport (Fig. 2B). Downregulation of *amyR* gene leads to lower secretion of glucoamylases, enzyme that cleaves maltose to glucose, resulting in downregulation of hexose transporters and reduced carbon source uptake by recombinant strains (Vongsangnak *et al*., 2009).

However, RESS mechanism details are still unclear as well as its specific targets. Zhou *et al*. (2014) described the octamer sequence TCACGGGC (positions −307 to −300) in the *amyB* promoter, as essential for downregulation under RESS induced by DTT. Based on these findings, we suggest that RESS is activated in our recombinant strains, which is consistent with previous observations.

#### The protein sequence context influences the secretion levels

As described above, the Anid_Cbhl strain secretes more recombinant proteins than Anid_AbfA (Fig. 1A). The overall response of both recombinant strains was quite similar; however, each strain showed an exclusive set of proteins more or less abundant (Fig. 4). Therefore, we analysed the sequences of AbfA and Cbhl enzymes. AbfA is 656 amino acids in length, with three cysteines predicted forming disulfide bonds and nine possible N‐glycosylation sites. Cbhl is 532 amino acids in length and has a predicted cellulose‐binding module corresponding to amino acid positions 500 to 528, twenty‐two cysteines predicted forming disulfide bonds and one possible N‐glycosylation site (Fig. S1).

Protein glycosylation is one of the most common forms of the post‐translational modification process, which are present across all kingdoms of life. N‐glycosylation, involved in the process of protein folding in the ER, plays an important role in biological activity. In several studies, the production of enzymes increased when the glycosylation‐related genes were introduced or its glycosylation sites changed (Han and Yu, 2015). However, the glycosylation role in recombinant protein production continues to be controversial. In *S*. *cerevisiae* N‐glycosylation‐deficient mutants, the expression and secretion of a *Bacillus licheniformis* thermostable α‐amylase were improved. The authors suggested that the transfer of oligosaccharides may compete with α‐amylase folding because of the slow transfer rates of the incomplete oligosaccharides. Folding preceded N‐glycosylation and resulted in an underglycosylation of the recombinant enzymes, which could be preferentially folded and secreted (Hoshida *et al*., 2013).

The secretion of human insulin precursor (IP), a small protein without glycosylation sites, was higher when compared to α‐amylase, a larger protein that has one N‐glycosylation site (Tyo *et al*., 2012). In *S. cerevisiae*, the disruption of genes involved in the N‐glycosylation modification improved the production of recombinant enzymes and the transcription of key genes in the folding pathway such as chaperones *KAR2*, homologous to BipA and other HSP70 chaperones (Tang *et al*., 2016). BipA and HSP70 were more abundant in the Anid_Cbhl strain, in which the recombinant protein production was higher than in the Anid_AbfA strain. This evidence suggests that the lower number of N‐glycosylation sites in cellobiohydrolase enhances recombinant protein production due to the improvement in the secretory pathway capacity.

The other bottleneck in recombinant protein production could be the odd number of cysteines in the AbfA. The random disulfide isomerization process may incorporate the cysteine that should not be incorporated into a disulfide bond, thereby generating futile cycles of disulfide formation. In α‐amylase and human insulin precursor production by yeast, the odd number of cysteines in α‐amylase was one factor contributing to the sixfold fewer molecules secreted when compared to insulin (Tyo *et al*., 2012).

## Experimental procedures

### *Aspergillus* strains and growth conditions

*Aspergillus nidulans* A773 (pyrG89; wA3; pyroA4) was obtained from the Fungal Genetic Stock Center (FGSC). *Aspergillus nidulans* A773 recombinant strains secreting high levels of GH51 α‐l‐arabinofuranosidase (Anid_AbfA) and GH7 cellobiohydrolase (Anid_Cbhl) and *A. nidulans* A773 transformed with empty vector (Anid_pEXPYR) were obtained from our culture collection maintained at the Institute of Biology, UNICAMP.

The Anid_AbfA, Anid_Cbhl and Anid_pEXPYR strains were constructed using the pEXPYR vector as expression plasmid. The pEXPYR is a shuttle vector used for expression and secretion of client proteins in *Aspergillus* species. pEXPYR contains ampicillin resistance marker for propagation in *Escherichia coli* and a phleomycin resistance eukaryotic selectable marker. For cloning and expression of proteins, target gene is overexpressed under control of the *A. niger* glucoamylase promoter and its N‐terminal secretion peptide and tryptophan synthase transcription terminator. Furthermore, the orotidine‐5′‐decarboxylase gene (*pyrG*) from *A. niger* present in this vector is useful to complement *A. nidulans* pyrG89 mutation in strains such as *A. nidulans* A773 (Segato *et al*., 2012b).

The heterologous genes (*abfA; cbhl*) were isolated from *Aspergillus fumigatus* by PCR, digested with NotI and XbaI and ligated onto NotI/XbaI digested pEXPYR plasmid. After cloning, the pEXPYR containing *A. fumigatus* genes and the empty pEXPYR was transformed into *A. nidulans* A773 (Damásio *et al*., 2012; Segato *et al*., 2012b).

The spores solution was inoculated in 15 ml of minimum medium (MM) supplemented with 2% (m/v) maltose as a gene expression inducer. The MM composition was 1× Clutterbuck's salts (20× Clutterbuck's salts stock: 1.4 M NaNO_3_, 0.13 M KCl, 0.042 M MgSO_4_·7H_2_O and 0.22 M KH_2_PO_4_), 1× trace elements (1000× Trace elements stock: 7.2 mM ZnSO_4_·7H_2_0, 17.7 mM H_3_BO_3_, 2.52 mM MnCl_2_·4H_2_0, 2.72 mM FeSO_4_·7H_2_0, 0.95 mM CoCl_2_·5H_2_0, 0.7 mM CuS0_4_·5H_2_0, 0.21 mM Na_2_Mo0_4_·4H_2_0 and 17.11 mM EDTA), 11 mM maltose, pyridoxine (1 mg l^−1^) and uracil/uridine (2.5 mg l^−1^). All experiments were carried out in three biological replicates.

### Maltose quantification by HPLC

Supernatant from *A. nidulans* cultures was collected after 12, 24, 48 and 72 h of growth, and the maltose concentration was detected using high‐performance liquid chromatography (HPLC) Agilent Infinity 1260 with a 50C IR detector, Aminex column HPX‐87H 300 mm × 7.8 mm at 50 °C and 0.5 ml min^−1^ of ultrapure Milli‐Q water as eluent phase.

### Samples preparation for liquid chromatography–tandem mass spectroscopy (LC‐MS/MS)

#### Intracellular proteome

The fungal mycelium was harvested after 24 h of growth and ground into a fine powder in liquid nitrogen. The mycelial powder was suspended in 10 volumes of extraction buffer in an ice bath (20 mM Tris pH8, 0.05% Triton, 150 mM NaCl and 2 mM PMSF), centrifuged at 7500 *g* for 10 min at 4 °C, and the supernatant was subsequently collected.

#### Extracellular proteome

Culture filtrates after 72 h of growth were washed with 2 ml ultrapure water and concentrated using 10 000 Da cut‐off Amicon. The samples were quantified using the Bradford method (Bradford, 1976), and ten milligrams of intracellular and extracellular proteins was loaded onto SDS‐PAGE. The band slices were destained with methanol and acetic acid, dehydrated with acetonitrile, reduced with DTT, alkylated with iodoacetamide and digested for 18 h with 20 ng μl^−1^ trypsin using an ammonium bicarbonate buffer. After digestion at 37 °C, the peptide extraction was carried out by methanol and acetic acid treatment (Shevchenko *et al*., 1996) with modifications.

### LC‐MS/MS analysis, protein identification and statistical analysis

The peptide mixture from the biological replicates was analysed by LTQ Velos Orbitrap mass spectrometer (Thermo Fisher Scientific, USA) coupled with liquid chromatography–tandem mass spectrometry using an EASY‐nLC system (Thermo Fisher Scientific).

The LC‐MS/MS raw files were used for the database search via the mascot software application (Matrix Science, London, UK), comparing the *A*. *nidulans* peptides from *Aspergillus* Genome Database (*Asp*GD) using a zero false discovery rate estimated by target/decoy searches. The Mascot was searched using a fragment ion mass tolerance of 0.80 Da and a parent ion tolerance of 15 PPM. S‐carbamoylmethylcysteine cyclization of the n‐terminus, oxidation of methionine, n‐formylation of the n‐terminus, acetylation of the n‐terminus, iodoacetamide derivative of cysteine and acrylamide adduct of cysteine were specified as variable modifications in Mascot.

scaffold (version Scaffold_4.2.1, Proteome Software, Portland, OR) was used to validate the MS/MS‐based peptide and protein identification. Peptide identifications were accepted if they could be established with a probability higher than 95% by the Peptide Prophet algorithm (Keller *et al*., 2002). Protein identifications were accepted if they could be established with a probability higher than 99.0% and contained at least two identified peptides. Protein probabilities were assigned by the Protein Prophet algorithm (Nesvizhskii *et al*., 2003). Proteome label‐free quantification and individual protein abundance were obtained using the total spectrum count method.

Functional annotation of the proteomes was performed using Fisher's exact test with a threshold of 95%. For the proteome data, we performed a statistical analysis (fold change; FC) and analysed the data using a paired *t*‐test with Bonferroni's post‐test for multiple comparisons, where only proteins with *P*‐values < 0.05 were selected. The resultant sequences were imported into FunCat for mapping the sequences into functional categories and a comparison was made between the proteomes from empty plasmid‐transformed strain (Anid_pEXPYR) and the proteomes from the *A. nidulans* recombinant strains (Anid_AbfA and Anid_Cbhl). Finally, intracellular and extracellular proteins were evaluated for the presence of a signal peptide (SP) or a secretion signal to the non‐classical pathway, which was performed using the SecretomeP server. Proteins with SP or those with a ‘threshold’ above 0.6 for non‐classical pathways were classified as extracellular (Bendtsen *et al*., 2004).

### RNA extraction, transcript analysis by qPCR (quantitative real‐time PCR) and primer design

To measure the α‐l‐arabinofuranosidase and cellobiohydrolase gene transcripts, mycelia of Anid_A773, Anid_pEXPYR, Anid_AbfA and Anid_Cbhl were harvested by filtration and used for RNA extraction. Harvested mycelia were ground into a fine powder in liquid nitrogen, and the total RNA extraction was performed using the RNAeasy mini kit (Qiagen) and then quantified using the gen5 software Take3 Sessions from Biotek Synergy HT spectrophotometer (Thermo Fisher Scientific). Synthesis of cDNA from total RNA was carried out using Maxima First Strand cDNA Synthesis Kit (Thermo Scientific) according to the manufacturer's instructions.

All the PCRs were performed using the QuantStudio 6 Flex Real‐Time PCR System (Solis BioDyne) and 5× HOT FIREPol Probe qPCR Mix Plus (ROX) (Applied Biosystems). Amplification reactions were performed in a final volume of 10 ul reaction mixtures containing 1× HOT FIREPol Probe qPCR Mix, 100–300 nM forward primer, 100–300 nM reverse primer and 100 ng cDNA templates. Real‐time PCR protocols were as follows: 12‐min initial denaturation at 95 °C, followed by 40 cycles of 5 s at 95 °C, 20 s at 60 °C. All analyses were conducted independently in triplicate with no amplification control (no added primers) and carried out in 96‐well plates, which were covered with optical tape. The specificity of PCR amplifications was documented by melting curve analysis. Transcript levels of *abfA* and *cbhI* genes were normalized, and the data analysis was performed using the ΔΔCT method and the relative standard curve method in according to the amplification efficiency of the targets. The primers used in real‐time PCRs are listed in Table S4.

## Conclusions

**Figure 6:**
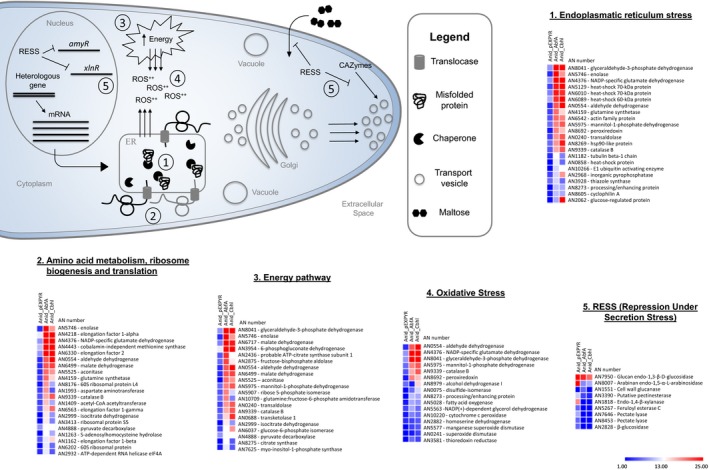
Overview of biological process overrepresented in *Aspergillus nidulans* recombinant strains. Heterologous protein production remains a complex process with some bottlenecks. Generally, the recombinant gene contains strong promoter for high level expression of the target mRNA. Large quantities of mRNAs overload the translational pathway, which increase misfolded proteins amounts in ER, inducing an ER stress (1). The homeostasis maintenance is achieved by UPR that induces genes coding to chaperones, amino acid metabolism, ribosome biogenesis, translation, among others (2). Furthermore, energy demand required for heterologous protein production increased (3), resulting in high levels of reactive oxygen species in the cell (4). The secretome analysis of recombinant strains showed the downregulation of biomass‐degrading enzymes and their genes, suggesting the presence of the RESS mechanism. Associated with the overload of misfolded proteins in the ER, this mechanism downregulates transcriptional activators, such as *amyR* and *xlnR* that regulates expression of several amylases and CAZymes respectively (5). The heat maps (at the bottom) represent the protein abundance in the recombinant (Anid_AbfA; Anid_Cbhl) and control strains (Anid_pEXPYR) in each biological process categorized by MIPS FunCat. The scale indicates the number of proteins found in each category.

The primary goal of this study was to analyse the intra‐ and extracellular proteome profiles of *A. nidulans* recombinant strains and to describe the major bottlenecks involved in the production of two different heterologous proteins. In this study, we identified that the intracellular profile of the recombinant strains Anid_AbfA and Anid_Cbhl is similar, despite producing different heterologous proteins. The Anid_Cbhl strain secretes more recombinant enzyme than Anid_AbfA, and we suggest that the higher amount of specific proteins such as PdiA, BipA, TrxA and AldA, which alleviate ER and oxidative stress, can contribute improving heterologous protein production. Moreover, we showed that the following processes – energy pathway, amino acid metabolism, ribosome biogenesis, translation, reticulum and oxidative stress were the main enriched mechanisms in the recombinant strains. The RESS phenomenon can be present in the recombinant strains, which probably prevents the high ER load with additional proteins during the high‐level production of heterologous proteins in *A. nidulans*. All these findings in recombinant strains are represented in Fig. 6.

In addition, we suggest that the context of the protein sequence directly impacts the difference in the heterologous protein secretion levels, evidenced by an odd number of cysteines and the number of N‐glycosylation sites. These findings helped us to comprehend the underlying mechanisms involved in the high secretion of recombinant proteins in *A. nidulans* and in the rational manipulation of target genes for the improvement of fungi strains as microbial cell factories.

## Conflict of Interest

None declared.

## Supporting information


**Fig. S1.** Overview of target proteins structure.

**Table S1.** List of proteins identified by LC‐MS/MS and spectrum counts on the replicates.

**Table S2.** Comparative analysis of proteins abundance in the recombinant strains.

**Table S3.** Total spectra of proteins identified in the *Aspergillus nidulans* secretomes.

**Table S4.** Oligonucleotides used in this study for qPCR analysis.
