# Supplementary material for: Protein profile in Aspergillus nidulans recombinant strains overproducing heterologous enzymes
Source: Microb Biotechnol. 2018 Jan 8;11(2):346–58. doi: 10.1111/1751-7915.13027 (PMC5812239; doi:10.1111/1751-7915.13027)
Supplement: Supplementary file 1 — Fig. S1. Overview of target proteins structure. [file MBT2-11-346-s001.pdf]

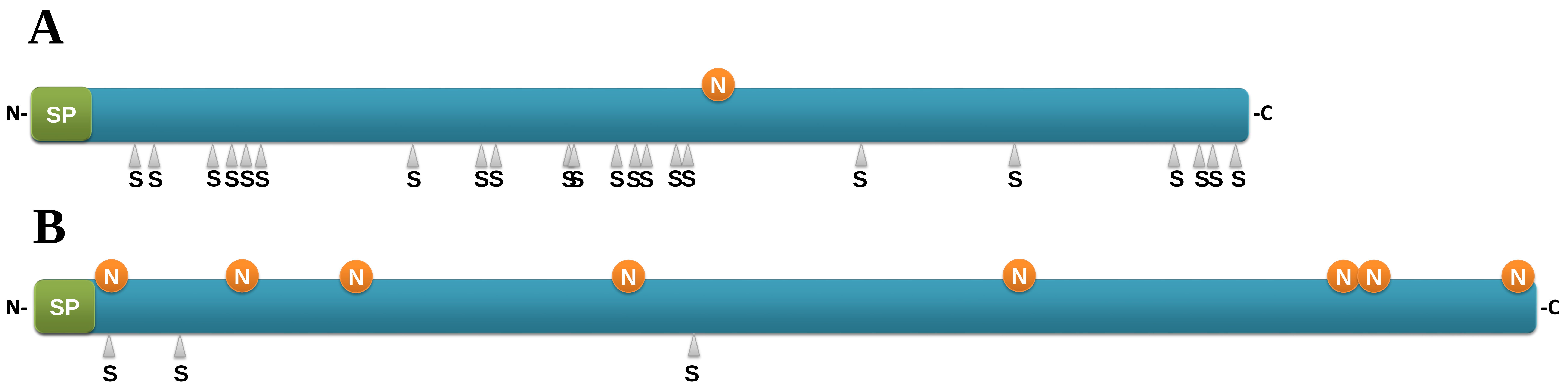

Fig. S1
