# Supplementary material for: Protein profile in Aspergillus nidulans recombinant strains overproducing heterologous enzymes
Source: Microb Biotechnol. 2018 Jan 8;11(2):346–58. doi: 10.1111/1751-7915.13027 (PMC5812239; doi:10.1111/1751-7915.13027)
Supplement: Supplementary file 5 — Table S4. Oligonucleotides used in this study for qPCR analysis. [file MBT2-11-346-s005.pdf]

Table S4: Oligonucleotides used in this study for qPCR analyses

Gene	Forward (5´to 3´)	Reverse (5´to 3´)
abfA (Afu2g15160)	TTCTGGGTGCCGAAAGAAAC	TGAGCATGGGTGCGTAGGT
cbhB (Afu6g11610)	GCATGACCGTCGACACCAAG	GGATCACCTTGCCGTTCTGC
tubC (AN6838)	ACTGCTCTGTGCTCTATG	TGTGAACTGGTTGGAGAC
xlnR (AN7610)	TCGGTGGTTTTGGTGTCTCG	GGCGACATCATCCACAGCTC
amyR (AN2016)	CCTCCCGTTCCATCTTCCTG	TGTTCCATCCCAATCCCATGC
